# Facial video photoplethysmography for measuring average and quasi-instantaneous heart rate: a pilot validation study

**DOI:** 10.3389/fphys.2025.1638809

**Published:** 2025-09-24

**Authors:** Leszek Pstras, Tymoteusz Okupnik, Beata Ponikowska, Bartlomiej Paleczny

**Affiliations:** ^1^ Nalecz Institute of Biocybernetics and Biomedical Engineering, Polish Academy of Sciences, Warsaw, Poland; ^2^ Department of Physiology and Pathophysiology, Faculty of Medicine, Wroclaw Medical University, Wroclaw, Poland

**Keywords:** remote photoplethysmography, vital signs, camera-based monitoring, contactless monitoring, pulse rate

## Abstract

**Background:**

Video photoplethysmography (vPPG) is a contactless optical technique for recording blood pulsations in the blood vessels of the skin using a digital camera that is increasingly used to measure or estimate various physiological parameters. In this study, we evaluated the accuracy of average and quasi-instantaneous heart rate (HR) measurements performed via facial vPPG technology Shen.AI Vitals and a smartphone camera.

**Methods:**

We studied 35 healthy volunteers in a seated position (median age 25 years, 17 females). Video recordings of participants’ faces were obtained using the front camera of a smartphone mounted on a tripod. In parallel, a 1-lead chest electrocardiogram (ECG) was recorded to obtain reference HR values (average value from the entire 60-s measurement and multiple values averaged over 10-s or 4-s periods during the measurement).

**Results:**

The mean absolute errors were 0.1, 0.2, and 0.4 beats per minute (bpm) for HR averaged over 60-s, 10-s, and 4-s periods, respectively. The errors did not exceed 1 bpm in 100.0%, 99.8%, and 94.5% of the cases, respectively. For the latter, our sample included almost 1,900 HR values from a relatively wide range (46–117 bpm). Regardless of the HR averaging time, the correlation between the vPPG-based and reference values was very strong (r > 0.99, P < 0.001).

**Conclusion:**

In predominantly young, white, seated subjects, the tested vPPG technology provided highly accurate HR measurements, both when the values were averaged over 60 s and in the case of short-term values averaged over 10 s or quasi-instantaneous values averaged over 4 s. To our knowledge, this is the first study on vPPG technology to examine quasi-instantaneous HR measurements (averaged over periods shorter than 5 s). The results should be confirmed in a larger study with greater diversity in age, skin tone, and lighting conditions.

## 1 Introduction

Heart rate (HR), or pulse rate, is one of the vital signs used in medical examinations for the basic assessment of the condition of the cardiovascular system. Resting HR can help detect various diseases and predict cardiovascular and all-cause mortality (Puig et al., 2022; Olshansky et al., 2023). In particular, a UK Biobank study of over 500,000 individuals followed for up to 12 years showed that a 10-bpm increase in resting HR was associated with 22% and 19% greater risks for all-cause mortality in men and women, respectively (Raisi-Estabragh et al., 2020). A meta-analysis including over 1.2 m individuals followed for up to 40 years showed that a higher resting HR was associated with increased cardiovascular and all-cause mortality, independent of traditional cardiovascular risk factors (Zhang et al., 2016). Moreover, an increase in the resting HR over time has also been shown to be associated with higher all-cause mortality, with the risk of death increasing by 33% for every 10-bpm increase over 6 years (Hartaigh et al., 2015). Therefore, regular HR assessment is an important part of prevention (Inoue et al., 2013; Jensen et al., 2021). HR also reflects the body’s response to stress, emotions, exercise, or other stimuli, making it useful for self-monitoring in health and wellness contexts (Reeder and David, 2016). It can also be used for monitoring the cardiac rehabilitation process (Dobrican and Zampunieris, 2016; Etiwy et al., 2019) as well as for monitoring fatigue and recovery process in athletes to prevent overtraining and optimize training effectiveness (Borresen and Lambert, 2008; Daanen et al., 2012; Buchheit, 2014). Moreover, it can be used to monitor drivers’ physiological state (Kuo et al., 2015; Zhang et al., 2017) or to monitor human-computer interactions (Przybylo, 2019).

Photoplethysmography (PPG), i.e., an optical technique for detecting blood pulsations in the skin vasculature, is commonly used for measurements or continuous monitoring of HR and other vital signs via special probes/clips typically attached to a finger or ear lobe (Allen, 2007). PPG sensors are also increasingly being integrated into a variety of wearable devices, such as smartwatches or wristbands (Bayoumy et al., 2021; Charlton et al., 2023). The two main limitations of classic PPG-based measurements are: 1) the need for a special device, such as a pulse oximeter or a wearable device equipped with PPG technology, which naturally limits the availability of such measurements, and 2) the need for skin contact, which may be important in healthcare facilities or in the case of measurements taken by another person or using someone else’s device, especially in the event of an epidemic.

The answer to the above limitations may be video PPG (vPPG), i.e., a remote PPG technique, also known as remote or imaging photoplethysmography, which uses digital video images of the skin to detect tiny changes in skin color caused by blood pulsations in superficial blood vessels and the resulting changes in the blood absorption of light incident on the skin (mainly by hemoglobin) (Molinaro et al., 2022). Such measurements can be performed using ambient white light (natural or artificial) as the source of light illuminating the skin, a consumer-grade camera integrated into a smartphone as the image sensor, and the smartphone processing power to analyze video images via a mobile app, thus making this technology accessible to most smartphone users without the need for any other device and without requiring skin contact. The possibility of using vPPG for contactless monitoring of HR and other vital signs has attracted much attention in recent years (Zaunseder et al., 2018; Chen et al., 2019; Hassan et al., 2020; Selvaraju et al., 2022; van Es et al., 2023), including the possibility of remote measurement of vital signs in telemedicine applications (Tohma et al., 2021), for patient triage purposes (Capraro et al., 2022; Caspar et al., 2021), or for monitoring drivers (Xu et al., 2023).

In this study, we investigated vPPG technology developed by MX Labs (Tallinn, Estonia), called Shen.AI Vitals. This technology uses face detection and tracking algorithms to obtain vPPG signals from several regions of facial skin during a 1-min video recording and then employs various signal processing algorithms to analyze and combine information from these signals (in the red, green, and blue channels) to estimate HR as well as other physiological parameters. In particular, two types of HR values are provided – after the measurement, the HR averaged over the entire 1 min is provided, whereas during the measurement (every 1 s), average values from shorter periods are provided, i.e., the average HR from the previous 10 s (default) or the previous 4 s (optional).

The aim of our study was to assess the accuracy and precision of HR measurements performed using the tested vPPG technology and a smartphone camera by comparing them with reference values obtained from a simultaneously recorded electrocardiogram (ECG). Specifically, we set out to examine not only the accuracy of measuring average HR, as is commonly done in vPPG studies (i.e., HR averaged over 1 min), but also HR averaged over shorter periods, including quasi-instantaneous HR measurements, i.e., HR averaged over just 4 s. To our knowledge, no previous vPPG studies have investigated the accuracy of HR values averaged over such short time windows (compared with ECG-based values).

## 2 Methods

### 2.1 Subjects

The study was carried out at the Department of Physiology and Pathophysiology of Wroclaw Medical University (WMU) in Wroclaw, Poland. We recruited 38 adult volunteers (students, employees, and collaborators of WMU) aged 20–43 years (median 25 years), of whom 20 were females. The subjects were generally healthy and in particular did not suffer from any cardiovascular disease. The exclusion criteria (which ultimately did not have to be applied) were as follows: arrythmia (other than sinus bradycardia or tachycardia), neurologic disorders in the form of spontaneous head tremor, inability to keep the head in the required position during measurement, respiratory disorders such as irregular or shallow breathing, facial shape deformation, or extensive damage, wound, burn, dressing or disease of the facial skin. The study was approved by the Bioethics Committee at WMU (approval number 227/2022), and written informed consents have been obtained from all study participants. Due to technical issues with some ECG signals (excessive noise or artefacts), we had to exclude data from three subjects, and hence the final analysis was based on data from 35 subjects (17 females). See Table 1 for the characteristics of the study participants.

**TABLE 1:** Characteristics of the study participants (n = 35). Continuous variables are presented as medians [interquartile range; full range].

Characteristic	Value
Number of males/females, n (%)	18/17 (51%/49%)
Age	25 [21–27; 20–42] years
Body mass	64 [57–75; 47–104] kg
Body height	174 [168–183; 160–193] cm
Systolic blood pressure^a^	115 [111–123; 100–140] mmHg
Diastolic blood pressure^a^	80 [75–86; 68–94] mmHg

^a^ average result of two brachial oscillometric measurements (before and after video recording) after one discarded measurement.

### 2.2 Study protocol

The subjects were asked not to engage in any strenuous physical activity before participating in the study. During the study, they remained seated for the entire time, with the back supported. To ensure that the measurements were taken under resting conditions, video recordings were started approximately 5 min after all data acquisition devices were connected and set up. Arterial blood pressure was measured twice before the video recording (at a 1 min interval) and once immediately after the video recording. The subjects were asked to remain steady during the video recording, to refrain from speaking, and to breathe normally. Each participant was measured once in a resting (sitting) condition. This measurement was followed by two other measurements (in other conditions or with a specific breathing pattern) that are beyond the scope of this study.

### 2.3 Tested parameters

We studied the following HR values provided by the Shen.AI Vitals technology on the basis of a 1-min facial vPPG measurement:

1. HR averaged over 1 min, i.e., the average HR from the entire measurement;
2. multiple HR values averaged over 10-s periods – these short-term average HR values (49 values per measurement) are calculated and provided every 1 s during the measurement (starting from the 11th second until the 59th second) based on the preceding 10-s period with a 1-s delay (e.g., at time t = 11 s, the calculated value corresponds to the average HR in the period between t = 0 and t = 10 s);
3. multiple HR values averaged over 4-s periods – these quasi-instantaneous HR values (54 values per measurement) are calculated and provided every 1 s during the measurement (starting from the 6th second until the 59th second) based on the preceding 4-s period with a 1-s delay (e.g., at time t = 6 s, the calculated value corresponds to the average HR in the period between t = 1 and t = 5 s);

### 2.4 Equipment

**FIGURE 1:**
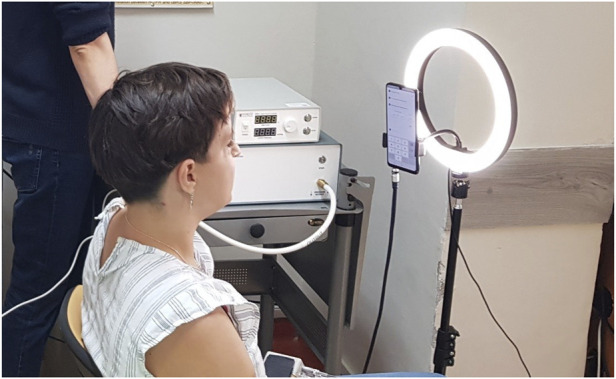
Location and setup of the measurements. The subject sat with the back supported. The phone was mounted on a tripod at head level with an LED lamp in the background.

Video recordings of participants’ faces were taken with the front camera of a mobile phone (Samsung Galaxy A22) mounted on a tripod at a distance of approximately 30 cm from the face (on average) and at a height adjusted to each participant (see Figure 1). The camera parameters were as follows: f/2.2, sensor resolution 13 MP, video resolution 1080p, and frame rate 30 fps. In parallel, a 1-lead chest ECG was recorded continuously via the Bio Amp ML132 module and the PowerLab data acquisition system (ADInstruments, Dunedin, New Zealand) with the following configuration of three electrodes (Einthoven lead I configuration): red (positive) on the right collarbone, white (negative) on the left collarbone, and black (reference) on the abdomen, to the left of the navel. Blood pressure was measured using a validated automatic upper arm blood pressure monitor (Omron M4 Intelli IT).

### 2.5 Lighting

The measurements were taken during the day, but considering that they took place in a laboratory located in the semi-basement, with occasionally limited access to daylight (especially under cloudy conditions) and potential shadows cast by nearby trees and/or vehicles passing on a nearby street, to keep the light conditions constant, we decided to have the window blinds closed and the ceiling lights on during all measurements, regardless of the time of day or weather. Additionally, to ensure a good level of light illuminating the participants’ faces, we used a ring-shaped LED lamp (with the light color corresponding to natural light) mounted on the tripod, with the phone in the center and in front of the lamp (see Figure 1). The intensity of light illuminating the faces of the study participants was around 1,700 lux (on average), as measured by a DT-1309 light meter (CEM Instruments, Shenzhen, China).

### 2.6 Video collection and processing

Video recordings were performed using a special mobile app developed and provided to us by MX Labs. This research app was designed to record a 2-min facial video that is then sent to the MX Labs cloud server to be processed and analyzed by the Shen.AI Vitals algorithms to provide estimates of various physiological parameters, including average HR from selected time windows of the recorded video. In the publicly available app from MX Labs (called Heart Monitor), the recording/measurement is limited to 1 min, and therefore, in the present study, all analyses were performed using only the first minute of the recorded video (thus ignoring the second minute). In the Heart Monitor app, all data processing and analysis takes place on the mobile device instead of on the cloud server, with the final results (including average HR) displayed to the user after the measurement and short-term HR values displayed during the measurement and updated every second. In our study, which employed the research app, both participants and laboratory staff were blinded to the results of the video measurements. These results (for all participants) were calculated on the MX Labs cloud server using the Shen.AI Vitals algorithms and provided to us by MX Labs only after the study was completed in digitalized tabular form. In this way, after calculating the reference HR values from the recorded ECG signals, we were able to compare the HR values obtained independently by the two methods. In particular, we were able to compare the multitude of short-term HR values, which would have not been possible if we were using the normal (non-research) app, where these values are displayed every 1 s during the measurement and are not available after the measurement. Moreover, in this research app, a special audio signal (a beep) was sent by the app to the PowerLab system at the beginning and end of each video recording to facilitate synchronization of the reference ECG signal with the video measurement, which was crucial for our study.

### 2.7 ECG processing

ECG signals recorded with the PowerLab data acquisition system (ADInstruments) were exported to LabChart 8 software (ADInstruments), which was used for automatic detection of QRS complexes and calculation of time intervals between successive heartbeats (R–R intervals). We used the LabChart default (human) settings for the QRS detection algorithm. In a few cases where automatic detection of the QRS complex failed, R peaks were marked manually in the LabChart interface. As mentioned earlier, in three subjects, the ECG signals were very noisy and contained various artefacts, which prevented the detection of most QRS complexes, and therefore, data from these subjects were excluded from the analysis.

The calculated R–R intervals (in ms) along with their time stamps (the times of their ends) were exported from LabChart to a text file, which was subsequently processed as follows using a script in MATLAB (MathWorks Inc., United States). First, we selected all R–R intervals that were entirely contained within the 60-s period corresponding to the first minute of the video recording (based on the aforementioned audio signal that was also exported to the same text file). Second, we corrected occasional wrongly identified R–R intervals (caused by falsely detected additional R peaks that were merely local noise, as confirmed by visual inspection of the ECG signals) – this was done by combining two adjacent R–R intervals whenever their total duration was between 80% and 120% of the median duration of all R–R intervals identified within the 60-s period of interest.

### 2.8 Calculation of reference HR values

The average HR (in beats per minute, bpm) was calculated as the reciprocal of the mean duration of all R–R intervals in the analyzed 60-s period. Similarly, short-term (10-s) or quasi-instantaneous (4-s) HR values were calculated as the reciprocal of the mean duration of all R–R intervals in the given period, i.e., in the previous 10 or 4 s, respectively, with a 1.3-s delay. For instance, reference 4-s HR average corresponding to the value provided by the tested technology at time t = 6 s was calculated as the reciprocal of the mean duration of all R–R intervals contained in the period between t = 0.7 s and t = 4.7 s. The additional 0.3-s delay was applied to account for the typical time shift between R-peaks in the ECG signal and the pulsation peaks in the facial vPPG signal, so that the R–R intervals used to calculate the reference short-term or quasi-instantaneous HR values matched the time intervals between the peaks in the vPPG signals considered by the tested technology. All reference HR values were rounded similarly to the tested values, i.e., to the nearest whole number.

### 2.9 Statistics

HR values obtained using the tested technology were compared with reference ECG-based values by calculating mean error (ME), standard deviations of errors (SDE), mean absolute error (MAE), and root-mean-square error (RMSE) in absolute terms as well as in relative terms, i.e., with respect to the reference values. 95% confidence intervals were calculated using the bootstrap method (with 10,000 samples). Additionally, for all analyzed HR parameters, the correlation between the tested and reference values was assessed using the Pearson correlation coefficient (r) and the agreement between them was visualized via scatter plots. Statistical significance was set at *P* = 0.05.

## 3 Results

According to ECG, the median value of the average HR among the study participants was 75 bpm (total range 52–98 bpm). In Table 2, we show various measures of accuracy and precision of HR values estimated by the tested vPPG technology (compared with ECG-based values), in both absolute and relative terms, i.e., with respect to reference values. Specifically, we assessed the average HR from the entire measurement (60 s) and multiple HR values averaged over shorter periods, i.e., 10 s (short-term HR) or 4 s (quasi-instantaneous HR). Regardless of the HR averaging time, the mean errors were close to 0.1 bpm. The mean absolute errors (MAEs) were approximately 0.1, 0.2, and 0.4 bpm for HR averaged over 60-s, 10-s, and 4-s periods, respectively. For the average HR from the entire measurement, there were no errors larger than 1 bpm (see Table 3). For short-term or quasi-instantaneous HR values, i.e., average values from 10-s or 4-s periods, the errors did not exceed 1 bpm in 99.8% and 94.5% of the cases, respectively. The maximal errors were 3 bpm for the 10-s averaging periods (1 case out of 1,715) and 4 bpm for the 4-s periods (4 cases out of 1,890).

**TABLE 2:** Measures of accuracy and precision of heart rate estimated by the Shen.AI Vitals vPPG technology (with 95% bootstrapped confidence intervals).

HR measure	Absolute errors (in bpm)			
	ME	SDE	MAE	RMSE
average HR (60 s)	0.09 [-0.03, 0.20]	0.37 [0.17, 0.49]	0.14 [0.03, 0.26]	0.38 [0.17, 0.51]
short-term HR (10 s)	0.12 [0.10, 0.14]	0.47 [0.45, 0.50]	0.23 [0.21, 0.25]	0.49 [0.47, 0.51]
quasi-instantaneous HR (4 s)	0.10 [0.07, 0.14]	0.78 [0.74, 0.82]	0.45 [0.42, 0.48]	0.79 [0.75, 0.83]

	Relative errors (as % of ECG-based values)			
	MPE	SDPE	MAPE	RMSPE
average HR (60 s)	0.11% [-0.04, 0.26%]	0.48% [0.22, 0.63%]	0.18% [0.04, 0.33%]	0.49% [0.22, 0.66%]
short-term HR (10 s)	0.16% [0.13, 0.19%]	0.62% [0.59, 0.65%]	0.30% [0.28, 0.33%]	0.64% [0.61, 0.67%]
quasi-instantaneous HR (4 s)	0.13% [0.09, 0.18%]	1.02% [0.97, 1.07%]	0.58% [0.54, 0.62%]	1.03% [0.98, 1.08%]

The errors between the vPPG-based and ECG-based heart rate (HR) values are expressed in absolute terms (in beats per minute, bpm) or in relative terms (in percentage of ECG-based values). Abbreviations: ME, mean error; SDE, standard deviation of errors; MAE–mean absolute error; RMSE, root-mean-square error, MPE, mean percentage error; SDPE, standard deviation of percentage errors; MAPE, mean absolute percentage error; RMSPE, root-mean-square percentage error.

**TABLE 3:** Distribution of absolute errors in heart rate (HR) values estimated by the Shen.AI Vitals vPPG technology. The vPPG-based HR values were compared with reference values obtained from simultaneously recorded electrocardiograms. The data show errors in HR averaged over 60 s as well as over shorter periods (10 s or 4 s).

Error	HR (60 s)	HR (10 s)	HR (4 s)
0 bpm	30 (85.7%)	1322 (77.1%)	1181 (62.5%)
1 bpm	5 (14.3%)	389 (22.7%)	605 (32.0%)
2 bpm		3 (0.2%)	79 (4.2%)
3 bpm		1 (0.1%)	21 (1.1%)
4 bpm			4 (0.2%)

Error	HR (60 s)	HR (10 s)	HR (4 s)
≤1 bpm	35 (100%)	1711 (99.8%)	1786 (94.5%)
≤2 bpm		1714 (99.9%)	1865 (98.7%)
≤3 bpm		1715 (100%)	1886 (99.8%)
≤4 bpm			1890 (100%)

**FIGURE 2:**
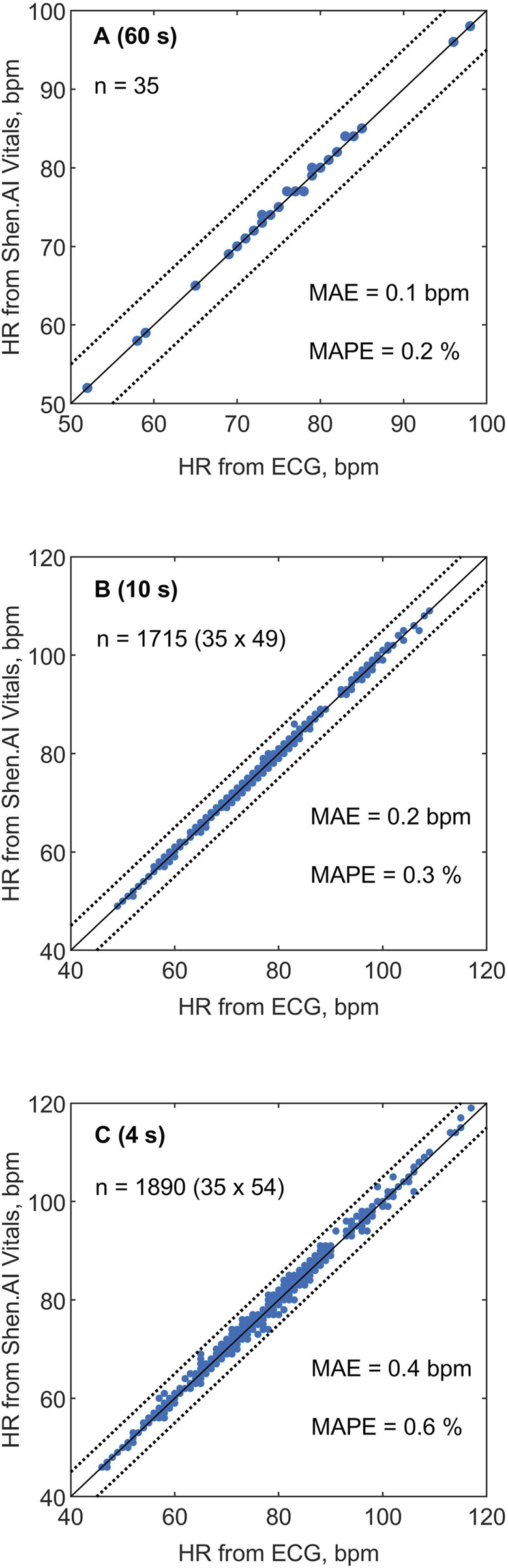
Heart rate (HR) values estimated in 35 subjects using the Shen.AI Vitals facial vPPG technology plotted against reference values obtained from simultaneously recorded electrocardiograms (ECGs). **(A)** Average HR values from 60-s measurements, **(B)** short-term HR values averaged over 10-s periods (49 values per subject), **(C)** quasi-instantaneous HR values averaged over 4-s periods (54 values per subject). The dotted lines indicate error limits of ±5 beats per minute (bpm). Abbreviations: MAE–mean absolute error, MAPE–mean absolute percentage error.

**FIGURE 3:**
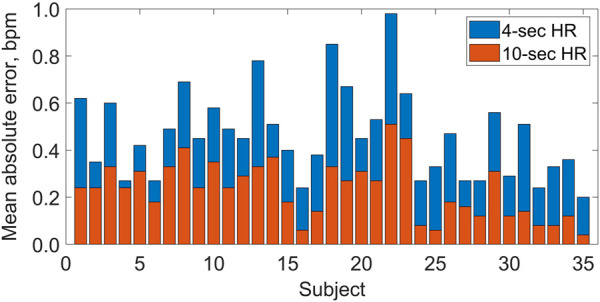
Mean absolute errors (per subject) in heart rate (HR) values estimated by the Shen.AI Vitals technology in multiple 4-s periods (blue bars, top) and 10-s periods (red bars, bottom) during 60-s video recording of the face, as compared with electrocardiogram-based values. For each subject, the two mean absolute errors correspond to the means of 54 and 49 values for HR averaged over 4-s and 10-s periods, respectively (see Methods for details). All bars start at 0.

Figure 2 presents the HR values obtained using the tested technology plotted against the reference values from ECG. The correlation between HR values from the two methods was very strong, as indicated by the Pearson correlation coefficients: r = 0.992 for the 60-s HR, r = 0.999 for the 10-s HR, and r = 0.997 for the 4-s HR (P < 0.001 in all cases). For individual subjects, the MAE varied between 0.2 and 1.0 bpm for the 4-s HR and between 0.1 and 0.5 bpm for the 10-s HR (see Figure 3), and the two MAEs were strongly correlated with each other (r = 0.817, P < 0.001).

**FIGURE 4:**
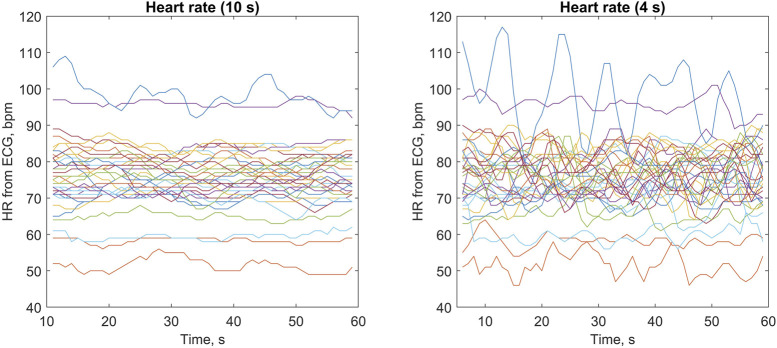
Heart rate (HR) values obtained from electrocardiograms (ECGs) averaged over 10-s periods (left panel) and 4-s periods (right panel) during 60-s measurements. Each line represents one of the 35 studied subjects (with the same color used in both panels), with HR values updated every 1 s (see Methods for details).

As shown in Figure 4 (right panel), the quasi-instantaneous HR values calculated from ECG (averaged over 4-s periods) showed different levels of variation during the 60-s measurements in individual subjects (e.g., related to breathing patterns). In most subjects, these quasi-instantaneous HR values varied within approximately ±5 bpm, with one subject showing particularly high HR variation (between 80 and 117 bpm). Across all subjects, we observed quasi-instantaneous HR values between 46 and 117 bpm. For HR values averaged over 10-s periods, as expected, the variation was accordingly lower, with low-frequency trends visible in some subjects (see the left panel in Figure 4).

## 4 Discussion

### 4.1 Key results

In this study on 35 predominantly young, healthy volunteers, we observed very high agreement between the HR values estimated by the tested vPPG technology via a smartphone camera and the reference ECG-based values, particularly for the average HR from the entire 60-s measurement, for which the errors did not exceed 1 bpm in all subjects, and in 86% of them there was virtually no difference between the vPPG-based and ECG-based average HR (both rounded to the nearest whole number). The mean absolute error (MAE) of these measurements was only around 0.1 bpm, whereas the root-mean-square error (RMSE) was around 0.4 bpm. Even for HRs averaged over much shorter periods, i.e., multiple HR values averaged over 10-s or 4-s periods, the agreement was also very high, with errors not exceeding 1 bpm in 99.8% and 94.5% of the cases, respectively, and the RMSEs of 0.5 bpm and 0.8 bpm, respectively. To our knowledge, this is the first study on the use of vPPG technology to evaluate the accuracy of quasi-instantaneous HR values (averaged over periods shorter than 5 s) compared to ECG-based values.

### 4.2 Standards

According to the standard ANSI/AAMI EC13:2002 (R2007) “Cardiac Monitors, Heart Rate Meters, and Alarms” (ANSI/AAMI, 2007), which was later superseded by ANSI/AAMI/IEC 60601-2-27:2011 (R2016) (ANSI/AAMI, 2016), the HR measurement error should not be greater than ±10% or ±5 bpm, whichever is greater. It should be noted, however, that this standard applies to ECG-based devices and it specifically excludes PPG-based devices. Nevertheless, given the lack of more appropriate standards, it is frequently used as a reference when assessing the accuracy of PPG or vPPG-based HR monitors (van Lier et al., 2020; Harford et al., 2019). In our study, we did not observe any errors in HR greater than 4 bpm or 10%, even for HRs averaged over 4-s periods, despite the relatively high variation in these quasi-instantaneous HR values during 60-s measurements. We investigated 1,890 such quasi-instantaneous HR values (54 in each of the 35 studied subjects) with a relatively wide range of values (between 46 and 117 bpm, according to ECG), and we observed errors of 4 bpm in only 4 cases (0.2%). These 4-s average HR values, which we call quasi-instantaneous, can be in fact treated as instantaneous HRs in line with the expert statement of the INTERLIVE Network regarding the validity of consumer wearable HR monitors, which indicates that instantaneous HR values should be averaged over time periods not longer than 5 s (Muhlen et al., 2021). The high level of accuracy of these (quasi-)instantaneous HR values observed in our study suggests that vPPG technology has the potential to be used in biofeedback applications (De Pascalis et al., 1991; Cengiz et al., 1997; Peira et al., 2013; Peira et al., 2014), although further studies are needed to generalize our findings to other populations and other conditions (see the Limitations section).

In a less strict standard, ANSI/CTA-2065 “Physical Activity Monitoring for Heart Rate and Related Measures” (CTA, 2018), developed by the Consumer Technology Association (CTA) for wearable devices for continuous HR measurements during physical activity, the accuracy criterion is that the mean absolute percentage error (MAPE) should not be greater than 10% for all measurements during the protocol proposed in that standard (pooled for all participants). Although we did not study a device for continuous HR measurements during physical activity, the short-term HR (averaged over 10 s) and the quasi-instantaneous HR (averaged over 4 s) are provided by the tested technology every 1 s, and hence, they can be treated as a kind of continuous HR measurements, which could last longer than the current measurement time (60 s). We observed MAPEs of only around 0.3% and 0.6% for HRs averaged over 10-s and 4-s periods, respectively, which are markedly lower than the level required by the above standard, although we emphasize that we studied sedentary and inactive subjects, which of course is of great importance here.

### 4.3 Time shift between the ECG and vPPG peaks

Note that for HR values averaged over 10-s and 4-s periods, the reference ECG-based values were averaged over the corresponding periods lagged by 0.3 s to account for the fact that peaks in the vPPG signals are delayed with respect to R peaks in the ECG used for heartbeat detection. For simplicity, in all subjects we used the same time shift of 0.3 s, which was approximately the typical time shift observed in our dataset. However, this time shift varied to some extent both between and within subjects (during the measurement). This means that in some cases, the reference ECG-based HR values could have been based on a slightly different number of heartbeat intervals, e.g., the number of R–R intervals in the given 4-s period could have been lower or higher by one compared to the number of peak-to-peak time intervals in the vPPG signal analyzed by the tested technology. We believe that this may at least partially explain some of the differences between the HR values estimated by the tested technology and the reference values, which could be avoided or at least mitigated by using personalized time shifts instead of the fixed value of 0.3 s (or even better, using dynamic time shifts). Therefore, we expect that the accuracy of the short-term or quasi-instantaneous HR values provided by the tested technology could be even higher, although even using a fixed time shift in our calculations, the observed errors are generally very low, and in particular lower than the maximum error levels required by the aforementioned standards. For the HR averaged over the entire measurement (60 s), we did not use any time shift when calculating the reference values from the ECG, as in this case such a time shift has a negligible impact. Again, taking into account such a time shift could lead to even better results in terms of accuracy of vPPG-based average HR values.

### 4.4 Previous studies

To the best of our knowledge, most data on the accuracy of vPPG-based measurements of HR are available for the Lifelight technology. In their largest study (VISION-D), which involved over 10,000 HR measurements in more than 5,700 subjects, the mean error (ME) was 0.3 bpm with a standard deviation of errors (SDE) of 4.0 bpm (Heiden et al., 2022), which was markedly higher than that in our study (0.1 and 0.4 bpm, respectively), although their study not only was much larger than ours but also included a very diverse population of patients and healthy volunteers, with a wide range of ages, health conditions, and skin tones, and a wider range of HRs (32–183 bpm). Note also that they used reference HR values from a sphygmomanometric measurement performed during a 1-min vPPG measurement, which on the one hand makes the comparison less reliable than when compared with ECG-based reference values, but, on the other hand, could explain greater errors. In a more recent, smaller study on the Lifelight technology, with 57 measurements in 19 participants and ECG-based reference HR values, the ME was higher in absolute terms (−0.6 bpm), but the SDE was lower (1.8 bpm) (van Putten et al., 2024), and hence, these measurements were more precise than those in their previously mentioned large study. In another study that investigated 1-min vPPG measurements of HR in 45 seated subjects (with relatively low variation in terms of age and skin tone), the MAE was reported to be 1.7 bpm, with an SDE of 2.1 bpm (Jain et al., 2016). In a study on 9 young, healthy volunteers with 8 measurements per subject, the results were only slightly worse than those in our study, with an ME of −0.2 bpm and an SDE of 0.6 bpm, compared with ECG-based values, although that study involved 2-min measurements with various breathing rates and depths (Shoushan et al., 2021).

With respect to shorter measurements, Hassan et al. compared several vPPG methods for 30-s measurements of HR in 45 healthy volunteers and obtained relatively high errors, with MEs ranging from 3.5 to 11.0 bpm and SDEs ranging from 3.5 to 8.2 bpm, although they studied measurements in a more natural environment, with uneven illumination of the participants’ faces and possible shadows or reflections, with a distance of 0.8 m between the camera and the face (Hassan et al., 2017). In a study on 25 subjects with 20-s vPPG measurements using a smartphone camera with a flash, Sanyal and Nundy reported an ME of −0.1 bpm and an SDE of 4.2 bpm (Sanyal and Nundy, 2018). In a study examining 10-s HR measurements (during 2-min video recordings) in 22 subjects, the ME was −0.2 bpm and the SDE was 1.8 bpm (Rivest-Hénault et al., 2021). Tran et al. investigated HR averaged over 8-s periods during 1-min video recordings in 10 subjects and reported an ME of −0.5 bpm and an RMSE of 5.7 bpm, although their dataset included measurements taken at various distances between the camera and the face, various lighting conditions, and intentional head movements (Duc Nhan et al., 2015). For an overview of the accuracy of vPPG-based measurements of HR, including older studies with low-resolution and/or low-frequency cameras, see the reviews by Rouast et al. (2018); Molinaro et al. (2022); Xiao et al. (2024).

### 4.5 Limitations

Our study had certain limitations. First, we studied a relatively small and homogeneous sample, which included only white subjects, and therefore our results are not necessarily generalizable to dark-skinned individuals, in whom PPG signals are usually of lower quality because more light is absorbed by the skin than reflected (Nowara et al., 2020; Fine et al., 2021). Second, we studied subjects in a sitting position who, in accordance with our request and requirements of the tested technology, kept their heads relatively still during the measurements and refrained from speaking. Although the tested technology includes a face tracking algorithm to account for possible head movements or changes in facial expressions, we have not investigated how these factors may affect measurement accuracy, which would require a separate study with intentional head movements and/or changes in facial expressions. Furthermore, our study used a single smartphone camera. Given that camera parameters, particularly sensor size and aperture, can significantly affect the amount of light collected by the camera (including the amount of light reflected from the face, as in our study) and therefore affect the quality of recorded vPPG signals, future studies should include measurements with different smartphone cameras with different parameters. However, the phone used in our study (Samsung Galaxy A22) was released in 2021, and therefore one should expect that newer phones will generally have higher quality cameras. This issue may therefore be more relevant when using cameras from other mobile devices, such as laptops, which generally have lower-quality cameras compared to smartphones. Moreover, our study was conducted in a laboratory setting, with controlled lighting conditions and the smartphone camera placed in a stable position at the participant’s head level. Although these conditions were similar to those typically observed in studies of this type, which facilitates comparisons of results across study participants, our results may not necessarily be generalizable to other, more natural settings. In particular, in our study the level of light illuminating the participants’ faces (on average around 1,700 lux) corresponded to a well-lit workspace for tasks requiring good visibility, such as detailed drawing or mechanical work. Therefore, the accuracy of the tested vPPG technology should be confirmed under lower facial illumination levels, i.e., lower than in our study but still sufficient to perform the measurement (with insufficient lighting, the quality of the vPPG signals will be too low and the user will be asked to improve the lighting conditions). On the other hand, higher illumination levels could potentially lead to better results (in outdoor conditions, the light intensity of 1,700 lux may correspond to midday of an overcast day, while on a bright, sunny day, the light intensity may exceed 100,000 lux). Finally, in our study the participants’ faces were illuminated relatively evenly. Although uniform facial illumination is one of the requirements of the tested vPPG technology, it may not always be easy to achieve in more natural conditions, and therefore the measurement accuracy should be further confirmed in studies with less even facial illumination.

## 5 Conclusion

In a group of predominantly young, white participants in a resting (sitting) position, the tested facial vPPG technology provided highly accurate heart rate measurements, both in terms of the average HR from measurements lasting 60 s as well as short-term HR values averaged over 10 s and quasi-instantaneous HR averaged over just 4 s, with errors not exceeding 1 bpm in 100.0%, 99.8%, and 94.5% of the cases, respectively. Our results support the feasibility of using a smartphone camera and facial vPPG technology to measure resting HR even over very short time periods, although this should be further confirmed in a study involving a larger group of participants with greater diversity in age, skin tone, and lighting conditions (both in terms of light intensity and uniformity).

## Data Availability

The raw data supporting the conclusions of this article (i.e., the anonymized heart rate data analyzed in the study) will be made available by the authors, without undue reservation.
